# Consultation on UTUC, Stockholm 2018: aspects of treatment

**DOI:** 10.1007/s00345-019-02811-w

**Published:** 2019-05-23

**Authors:** Helene Jung, Guido Giusti, Harun Fajkovic, Thomas Herrmann, Robert Jones, Michael Straub, Joyce Baard, Palle Jörn Sloth Osther, Marianne Brehmer

**Affiliations:** 1 Department of Urology, Urological Research Center, Lillebaelt Hospital, University of Southern Denmark, Vejle, Denmark; 2 Department of Urology, IRCCS San Raffaele Hospital, Ville Turro Division, Milan, Italy; 3 Department of Urology, Medical University of Vienna, Vienna, Austria; 4 Department of Urology, Kantonsspital Frauenfeld, Frauenfeld, Switzerland; 5 Institute of Cancer Sciences, University of Glasgow, Glasgow, Scotland UK; 6 Department of Urology, University Hospital Klinikum rechts der Isar, Technical University Munich, Munich, Germany; 7 Department of Urology, Amsterdam UMC, University of Amsterdam, Amsterdam, The Netherlands; 8 Division of Urology, Department of Clinical Sciences, Danderyd Hospital, Karolinska Institutet, Solna, Sweden

**Keywords:** Urothelial cancer, Upper urinary tract, Radical nephroureterectomy, Kidney-sparing surgery, Adjuvant treatment, Mitomycin C, BCG instillation, Chemotherapy, Lymph node dissection

## Abstract

**Purpose:**

To provide an overview of treatment modalities for management of upper tract urothelial carcinoma (UTUC).

**Methods:**

In accordance with the standards for a scoping review, data presentation and discussion at the Consultation on UTUC in Stockholm, 6–7 September 2018, consensus was reached on the latest and most important treatment recommendations for UTUC. Using Pubmed, Web of Science, and Embase, publications were selected based on quality, clinical relevance, and level of evidence.

**Results:**

Kidney-sparing surgery should be attempted for low-grade UTUC. Radical nephroureterectomy with bladder cuff excision is first option for high-grade disease. Post-operative bladder instillation of chemotherapy should be offered after RNU to reduce intravesical recurrence rate. Identification of tumor grade and stage is crucial when selecting treatment. Ureteroscopic management of low-grade and non-invasive UTUC achieves disease-free survival similar to that offered by radical nephroureterectomy but seems to be a risk factor for intravesical recurrence. Lymphadenectomy appears important for high-risk disease, although the therapeutic benefit needs further validation. There is little evidence supporting use of Bacillus Calmette–Guérin (BCG) and mitomycin C as monotherapy and adjuvant treatment in UTUC. A randomized clinical trial has indicated that platin-based chemotherapy for invasive UTUC improves disease-free survival, suggesting that adjuvant chemotherapy should be considered standard care for ≥ T2 N0–3M0 disease.

**Conclusions:**

Risk stratification assessment is feasible and mandatory in UTUC. Identification of tumor grade and stage is essential for optimal treatment selection. Kidney-sparing surgery should be offered in low-risk disease, whereas radical nephroureterectomy and adjuvant chemotherapy should be considered in high-risk disease.

## Introduction

The EAU guidelines for treatment of upper tract urothelial carcinoma (UTUC) have evolved. RNU was the gold standard for all cases until 2013 but is recommended only for high-risk tumors since 2015 [1]. KSS is the preferred strategy for management of all low-risk UTUC in the 2017–2018 edition. UTUC is relatively rare and due to the scarcity of prospective and randomized controlled studies (RCS), the evidence and grades of recommendations are limited regarding diagnostic and treatment procedures. The aim of Consultation on UTUC 2018 was to gather clinical and research experts in diagnostics and treatment of UTUC to discuss existing guidelines, and, by reviewing the literature, to add further recommendations. The experts were assigned different topics regarding UTUC. Due to the lack of RCSs, they were instructed to investigate the topic in accordance with a scoping review.

## Treatment selection

The 2018 EAU guidelines recommend that endoscopic ablation be considered in low-risk cancers, whereas RNU is the first option in cases of organ-confined high-grade disease.

Tumor classification based on pathological grade and stage is a key point in treatment selection. Clinically, it is difficult to determine tumor stage in UTUC, and thus risk stratification of low- and high-risk tumors [1] is useful for identifying patients who are more suitable for KSS than for RNU. To define low-risk and high-risk UTUC, the cytological and histological grades of the tumor cells are the most essential factors [2, 3] although tumor size and multifocality also should be taken into consideration.

Grasso et al. [4] analyzed the outcome in 160 consecutive patients undergoing either ureteroscopic treatment or extirpative RNU for UTUC. These investigators concluded that tumor grade was the most significant predictor of both overall survival (OS) and cancer-specific survival (CSS), regardless of treatment method. In a systematic review of the oncological outcomes of ureteroscopic or percutaneous treatment of UTUC [5], it was concluded that the rate of recurrence is high in endoscopically managed UTUC, and a grade-related risk of tumor progression and disease-specific mortality were also documented. The review further indicated that, for highly selected low-grade tumors, local endoscopic treatment might be comparable to RNU in terms of 5-year disease-specific survival (DSS). For high-grade disease, DSS was poor, and endoscopic management should only be considered for compelling imperative indications, including solitary kidney or severely impaired renal function.

Although there seems to be consensus regarding the importance of tumor grading, correct grading is challenging [6–8].

### Kidney-sparing surgery (KSS) for UTUC

Focal treatment of UTUC includes endoscopic therapy: ureteroscopic or percutaneous; ureteral segmental resection; and local instillation of Bacillus Calmette–Guérin (BCG) vaccine or mitomycin C.

In patients with low-risk disease, survival rates after KSS are comparable with those after RNU, however, with lower morbidity and without kidney function loss [9]. The 2010 United States Renal Data System (USRDS) Annual Data Report pointed out that 5-year overall survival for end-stage renal disease was only 39% and argued that KSS should also be considered for high-grade tumors after frank patient counseling in special, imperative cases [10].

### Management of UTUC by ureteroscopy (URS)

In 2017, the International Consultation on Urologic Diseases (ICUD) reported an update on focal treatment of low-risk UTUC [11], stating that no prospective randomized studies were found to support surgical management guidelines. Twenty-one different series of ureteroscopic treatment (1989–2014) were reviewed, and the overall survival rates varied from 35 to 100% (median follow-up 14–32 months). In a cohort study of 15 patients who underwent endoscopic laser ablation as primary treatment for low-grade UTUC, the recurrence rate was 33% within a follow-up period of median 25.5 months (range 13–51 months) [12]. The renal preservation rate was 80%. A relatively high incidence of recurrences emphasizes the importance of stringent follow-up in patients treated endoscopically. In an assessment comparing the outcomes of endoscopic management of non-invasive T1 and RNU for UTUC [13], the DSS during 50 months of follow-up was equivalent for the two methods. However, RNU was significantly superior to endoscopy for T2 and T3 tumors, with DSS of 91.7% vs 62.5% for T2 lesions, and progression-free survival (PFS) of 88.9% vs 55.6% for T3 tumors.

In a retrospective study of 41 UTUC patients, Villa et al. [14] evaluated the cancer detection rate of a second-look URS performed within 60 days of the first URS with laser ablation. The cancer detection rate at second-look URS was 51.2%, which emphasizes the necessity of a second look at 6–8 weeks and a stringent follow-up. In a meta-analysis examining the impact of URS before RNU on oncological outcomes [15], patients with initial URS had a significantly higher risk of bladder recurrence; however, that had no impact on CSS, OS, recurrence-free survival (RFS), or metastasis-free survival (MFS). Several options are available for laser ablation, such as holmium, thulium, and neodymium, but there is little evidence regarding the pros and cons of the different modalities [16].

### Management of UTUC by percutaneous access

Percutaneous access can be an option in cases with difficult retrograde access, e.g. due to upper urinary tract anomalies, urinary diversions or strictures. However, percutaneous access entails the risk of tumor seeding. The evidence base for percutaneous management is low, because evaluations in this area have entailed small case series with variability in duration of follow-up and measures of oncological outcome. A systematic review of percutaneous and ureteroscopic management of UTUC indicated that DSS in patients with T1 tumors was similar for the two approaches (89% vs 91%) [5]; the 5-year DSS was 81–100% for low-grade disease and ranged from 69 to 86% for high-grade disease. The ICUD group reviewed ten different series of percutaneous access treatments for low-risk UTUC performed over the period 1992–2015 [11], which showed CSS ranging from 75 to 100% and kidney-preserving rates of 65–94%.

### Management of UTUC by segmental ureteral resection

Segmental ureteral resection (SU) can be considered in selected patients with localized high-grade and invasive UTUC, requiring kidney-sparing management due to impaired renal function or solitary kidney. With this approach, oncological outcomes equivalent to those observed after RNU have been observed [9]. Lymphadenectomy is feasible during SU, but staging prior to surgery is of great importance [17]. A meta-analysis including 3963 UTUC patients supported the equivalence between SU and RNU in terms of CSS, OS and RFS [18]. However, patients treated with SU were selected for favorable features and the rate of positive lymph node disease was significantly lower in this group. Patients receiving SU had significantly better renal function preservation and the authors suggested SU as first-line treatment in selected cases of high-risk disease.

## Radical treatment of UTUC

### Indications

A tendency toward overtreatment was noted in a study of 2244 patients who underwent RNU [19], showing that 25% of the patients had pT0, pTa, or pTis tumors, and 18% low-grade tumors. RNU as first-line treatment for high-risk UTUC is challenged by the risk of chronic kidney disease. In a study of 336 UTUC patients, Lane et al. [20] determined the eligibility for cisplatin-based combination chemotherapy (CBCC) by measuring the estimated glomerular filtration rate (eGFR). The results showed that RNU eliminated CBCC as an adjuvant therapy option in 49% of high-risk patients, and only 22% of patients were eligible for post-RNU CBCC due to a decline in eGFR. The authors suggested that this problem might be resolved by applying multimodal treatment paradigms, with a focus on neoadjuvant chemotherapy.

### Radical nephroureterectomy (RNU) with bladder cuff excision

RNU with bladder cuff excision is the standard treatment for high-risk organ-confined UTUC, regardless of tumor location [1]. Due to the risk of tumor recurrence in the distal ureter and its orifice, the bladder cuff must be resected in connection with RNU. Several techniques are used for this purpose: endoscopic, transvesical, and extravesical approaches. A retrospective study evaluating recurrence and survival after laparoscopic nephroureterectomy (LNU) using either transvesical cystoscopic secured detachment and ligation or extravesical laparoscopic stapling found poorer RFS for patients treated with the latter method [21]. In a retrospective analysis of 2681 RNU-treated UTUC patients [22], endoscopic resection of bladder cuff, using transurethral resection with a hook-electrode, resulted in intravesical recurrence in a significantly larger number of patients than transvesical or extravesical approach. Hence, it was recommended that this approach be avoided.

In a retrospective analysis of 324 UTUC patients treated with RNU, 1995–2008, open radical nephroureterectomy (ORN) was compared to LNU with regard to RFS and DSS [23]. Two-year RFS was found to be similar in the two groups: 38% and 42%, respectively. It should be noted that lymph node dissection (LND) was performed in 81% of the ORNs but in only 70% of the LNUs. The data from that retrospective evaluation were confirmed in a cohort study published in 2011 [24], which showed oncological equivalence between ORN and LNU with regard to both RFS and CSS. Another investigation including 140 UTUC patients [25] reported that surgery duration was significantly longer for LNU than for ONU (240 vs 190 min.), but DSS did not differ significantly between the two methods. In a study of 80 patients with non-metastatic UTUC [26], MFS and CSS were equivalent for LNU and ORN; however, when matching for T3 and high-grade tumors, CSS and MFS were statistically higher for ORN. Based on these results, ORN was suggested as first-option treatment for patients with advanced stage disease. Retroperitoneal metastatic dissemination and metastases along the trocar pathway have been reported in up to 2.8% after LNU [27], and hence precautions should be taken to avoid spillage during pneumoperitoneum [1].

### Lymph node dissection in UTUC

Evidence is limited regarding the therapeutic advantage of LND in UTUC. The optimal lymph node template and the exact oncological advantages of LND remain to be defined. Although an increased trend towards LND in RNU, 64% of 1512 RNU-treated UTUC patients did not receive concomitant LND [28]. Of patients treated laparoscopically, only 24% had a lymph node dissection and had significantly fewer lymph nodes removed compared to patients treated by open RNU. Studies have indicated that LND involves a staging benefit by providing prognostic measures valuable for CSS. Roscigno et al. [29] concluded that nodal status was a significant predictor of CSS, and that pNx was associated with a worse prognosis than pN0 for T2–T4 tumors: 5-year CSS rates were 35%, 69%, and 77% for N +, Nx, and N0 diseases, respectively. Kondo et al. [30] conducted a prospective non-randomized study to compare the oncological outcomes of UTUC (pT2 or more) in 77 patients treated with both RNU and LND, and 89 treated with RNU only. CSS was significantly higher in the group treated with both RNU and LND (89.8% vs 48%). This study also documented an important difference between renal pelvic and ureteral UTUC, in that patients with the latter disease did not benefit from LND in terms of CSS.

In 2007, Suttman et al. [31] published their conceptual evaluation of the fragility of LND as a therapeutic tool in urothelial cancer. This assessment highlighted the principles of stage migration in radical cystectomy with LND for urothelial muscle-invasive bladder cancer (MIBC), and the investigators questioned the therapeutic benefits of that approach. In a randomized prospective trial, Gschwend et al. [32] showed that extended removal of lymph nodes and radical cystectomy in invasive urinary bladder cancer did not reduce the rate of tumor recurrence in the expected range.

A translational study by Marits et al. [33] showed that tumor-draining nodes in urothelial cancer can be considered to be a part of the immunological defense against urothelial cancer. This was demonstrated as anti-tumor-specific T cells being upregulated in tumor-draining sentinel nodes but not in non-draining lymph nodes. Also, the phenomenon of a T-cell line of defense has recently been further examined in MIBC patients receiving neoadjuvant chemotherapy [34], and the results illustrate the potential importance of an intact set of active regional immunological defense cells.

Kondo et al. [35] showed that in pT3 or more advanced urothelial cancer, the extent of LND has a significant impact on CSS (all cases N0). In accordance with these findings, Roscigno et al. [36] observed longer survival in N0 patients, who had at least eight lymph nodes removed during RNU for UTUC, whereas neither RFS nor CSS was associated with the extent of LND in N + patients. In contrast, an additional investigation by Kondo et al. [37] showed that the particular template of LND, not the number of lymph nodes removed, influenced CSS in 80 UTUC patients with T2 or > N0, which suggests that the extent of LND should be determined by the template alone, not necessarily by the number of lymph nodes removed.

In summary, it seems that for patients with a high-grade tumor, a large tumor burden, and local invasion, LND is advantageous in terms of improving staging accuracy and can thereby serve as a counseling amendment in the individualized follow-up scheduling. It is also plausible that LND can be curative in a subpopulation with limited nodal disease, but such a therapeutic benefit remains to be evaluated. Moreover, it appears that the anatomic extent and completeness of LND is an important aspect, although the chiefly retrospective data on the indications and accurate template for LND require further validation.

### Instillation therapy with BCG and mitomycin C: monotherapy, adjuvant therapy, and the role of bladder instillation in UTUC management

#### Bladder instillation therapy

The risk of bladder recurrence after RNU for UTUC is 22–47% but can be reduced by introducing intravesical chemotherapy (mitomycin C) [38, 39]. The beneficial effects of intravesical therapy have been documented in two randomized clinical trials (RCTs) and a meta-analysis [38–40], showing that a single dose of mitomycin C administered within 72 h after RNU resulted in a 52% reduced risk of bladder tumor recurrence within the first post-operative year.

### Instillation therapy in the upper urinary tract

Local recurrence rates as high as 70% have been observed in studies evaluating the efficacy of ureteroscopic treatment of UTUC [41]. A systematic review performed by Cutress et al. [5] found a 52% recurrence rate after endoscopic treatment. Instillation of BCG vaccine or mitomycin C in the upper urinary tract via a percutaneous nephrostomy tube or a ureteral stent can be used as adjuvant therapy after KSS for Ta/T1 upper tract tumors or for treatment of carcinoma in situ [42, 43]. However, no RCTs have assessed such treatment, and thus the level of evidence is low. A review evaluating the outcomes of studies using topical adjuvant BCG for upper tract carcinoma in situ documented an initial positive response of 73%, a recurrence rate of 26%, and a progression rate of 14% [5]. The follow-up time varied from 20 to 51 months. Complications reported included hematuria, pyrexia, fever, LUTS, septicemia (one fatal), and ureteral stricture.

### Retrograde instillation via ureteral catheter

The most advantageous and reliable approach to access the urinary tract for instillation therapy has been discussed. Liu et al. [44] used a fluorescent dye solution to examine three different modes of delivery in a pig model. Compared with antegrade perfusion and vesico-ureteral reflux via a ureteral stent, applying retrograde infusion via an open-end ureteral catheter resulted in the highest staining intensity in all six pre-defined points in the urinary tract. Pollard et al. [45] had similar results in an ex vivo porcine model. Retrograde infusion through an open-ended ureteral catheter resulted in a stained surface area of 83.6% compared with areas of 65.2% and 66.2% after delivery via an antegrade nephrostomy tube and reflux delivery through a JJ stent, respectively (*p* = 0.002). Clinical studies have not been conducted to confirm these experimental observations, and the clinical and pathophysiological consequences of high-volume infusion of chemotherapy have not been taken into consideration. High inflow pressure through the ureteral catheter might be necessary to reach all parts of the calyx system. Previous clinical studies of the pressure–flow relationship in the urinary tract have indicated that very high non-physiological pressure levels may be reached [46, 47], which may result in adverse effects such as infections and systemic loading with the installation substances as a result of intrarenal and pyelovenous backflow. Retrograde instillation should be used with extreme caution due to the potential risk of ureteral obstruction and subsequent pyelovenous backflow. Moreover, the risks associated with increased intrarenal pressure (i.e., infection and urosepsis) should be taken into consideration [46, 47].

### Antegrade instillation via a nephrostomy tube

In a retrospective study including 64 renal units, antegrade BCG treatment was given with curative intent in 42 cases and with adjuvant intent in 22 cases [43]. During a mean follow-up of 42 months, local recurrence was observed in 47% of cases. It seemed that better local disease control was achieved in patients treated with curative intent for Tis than in those treated adjuvantly for Ta/T1. In general, the treatment was well tolerated, although adverse events, mostly minor (fever, lower urinary tract symptoms, hematuria, mild infection), occurred in 20% of patients. There was one case of fatal *E. coli* septicemia, which highlights the importance of maintaining low intrarenal pressure [46, 47].

Knoedler et al. [48] reviewed data from studies reporting upper urinary tract instillation therapy performed with curative intent in patients with Tis UTUC. The most widely used medical agent was BCG; approaches were antegrade or retrograde, or a combination of the two, and resulted in response rates of 60–80%. Metcalfe et al. [49] investigated the efficacy, safety, and tolerability of mitomycin C induction and maintenance adjuvant topical therapy in 27 endoscopically treated patients with primary Ta/T1 UTUC. During a median of 19 months of follow-up, 60% of the patients were recurrence free, 80% progression free, and 76% RNU free. The 3-year OS rate was 92.9%. A variety of complications were observed, including recurrent urinary tract infection, severe bladder spasms, ureteral stricture, and pyelonephritis.

## Future options

Drug-eluting biodegradable stents and sustained-release mitomycin gel have been suggested as new treatment options for topical instillation therapy. The mitomycin gel functions as a liquid thermosensitive polymer at low temperatures but forms a gel at body temperature, resulting in prolonged retention and slow, sustained release of the therapeutic agent [50]. Barros et al. [51] developed a biodegradable stent that achieved 100% release of an impregnated anticancer drug in an artificial urine solution within 72 h. The development of such devices may have advantages in future treatment of UTUC.

## Systemic therapies

Both similarities and differences are apparent when comparing bladder cancer and UTUC. Although there is consistency between histological findings in the two diseases, the certainty of pre-operative staging and the availability of pre-operative histology are more complicated in UTUC. Platinum-based combination chemotherapy is expected to be effective in UTUC, but not all patients are eligible for such treatment due to comorbidity and impaired renal function after RNU.

Immune checkpoint inhibitors, which have favorable safety and anti-tumor activity profiles, have paved the way for a new era in the treatment of advanced UC. The primary molecular targets for these inhibitors are the programmed cell death-1 (PD-1) and programmed death-ligand 1 (PD-L1) checkpoints, which act as co-inhibitory signals that block anti-tumor effector T-cell responses [52]. Atezolizumab is a fully humanized monoclonal antibody of IgG1 isotype that selectively binds to PD-L1 and thereby enables T cells to overcome peripheral tolerance against tumor cells. In the IMvigor210 trial [53], atezolizumab showed durable activity and good tolerability in patients who had inoperable locally advanced or metastatic urothelial carcinoma that had progressed after previous platinum-based chemotherapy.

To define prognostic relevance of the primary location of urothelial carcinoma with regard to survival of the patient, the European Organization for the Research and Treatment of Cancer (EORTC) conducted a retrospective analysis of prospectively collected data, from three investigations of urothelial carcinoma [54]. Patients were grouped by primary tumor location (bladder cancer [*n* = 878] vs UTUC [*n* = 161]). The bladder cancer patients had better performance status. However, it was concluded that primary tumor location had no impact on PFS or OS in patients receiving platinum-based combination chemotherapy. The oncological outcomes for metastatic disease were similar in the two groups.

Patients with advanced urothelial carcinoma progressing after platinum-based chemotherapy have a poor prognosis and limited treatment options. In a study of patients with platinum-refractory advanced urothelial carcinoma [55], treatment with the highly selective monoclonal antibody pembrolizumab was associated with longer OS (3 months) and a lower rate of adverse effects compared with chemotherapy as second-line treatment. The median OS was 10.3 months for the total population of 542 patients.

### Adjuvant chemotherapy

Only a few studies have focused on systemic chemotherapy in locally advanced UTUC. In a retrospective investigation of 43 UTUC patients with T2 or more advanced M0 disease, Kwak et al. [56] evaluated the effect of cisplatin-based chemotherapy following RNU. Thirty-two patients received chemotherapy, whereas 11 declined such treatment. After 30 months of follow-up, the disease-free survival (DFS) was 63.6% in the chemotherapy group compared to 37.5% in the surveillance group. Adjuvant chemotherapy was not associated with any survival benefit in 312 pT2-4N0/x patients treated for UTUC with RNU [57]. In this retrospective study, the most detrimental effects of chemotherapy were seen in patients with Nx or N0 disease. Such retrospective case series remain prone to selection bias and, thus, call for more randomized, prospective trials with clearly defined patient selection criteria.

The UK National Cancer Research Institute conducted a phase III randomized trial of peri-operative chemotherapy vs surveillance in UTUC (designated the POUT trial) (NCT01993979). Eligible for inclusion were RNU-treated patients with pT2–pT4 N0M0 or pTany N1–N3M0 UTUC and a performance status of 0–1. Furthermore, GFR was to exceed 30 ml/min, and patients with significant comorbidity were excluded. The planned sample size was 383 patients. The patients that were included were randomized to either surveillance or platinum-based chemotherapy within 90 days following RNU. In the chemotherapy group, all patients received gemcitabine, and either cisplatin or carboplatin. Carboplatin was permitted only if the GFR was 30–49 ml/min. The primary endpoint of the POUT trial was DFS, and the secondary endpoints were MFS, OS, toxicity, treatment compliance, and quality of life. Publication of the final results is eagerly awaited, but a provisional report has announced that the trial has met its primary endpoint demonstrating improved disease-free survival for patients who commence adjuvant chemotherapy within 90 days of nephroureterectomy (Birtle et al. Abstract 407; ASCO GU meeting, San Francisco, 2018).

### Neoadjuvant chemotherapy

Neoadjuvant chemotherapy (NAC) for advanced UTUC is challenging due to the difficulties in pre-operative histological staging. Limited retrospective data support neoadjuvant chemotherapy prior to RNU, but survival data must mature to yield valid information in this area [58]. A retrospective study of 234 patients with cT3–4 or cN + disease concluded that the 101 patients receiving neoadjuvant chemotherapy (cisplatin or carboplatin) had an improved RFS and CSS compared to the 133 patients that had no NAC. The overall survival was, however, not improved by NAC [59]. This is in opposite to findings by Porten et al. who reported improved OS and DSS for 31 patients with high-risk UTUC receiving cisplatin-based NAC [60]. Likewise, Hosogoe et al. reported improved oncological outcomes for 51 pair-matched patients (≥ T3 or N +) receiving cisplatin plus gemcitabine prior to RNU [61]. However, retrospective case series are highly prone to selection bias and non-randomized trials do not report true clinical benefit. To date, there are no randomized prospective trials of neoadjuvant systemic therapy in UTUC.

## Conclusions

Identification of tumor grade and stage is essential for optimal treatment of UTUC. RNU is the first option for patients with organ-confined high-grade disease, whereas KSS should be considered for those with non-invasive low-grade UTUC. Laparoscopic and open RNU are both acceptable techniques. However, in stage pT3 or more, open RNU is recommended. Post-RNU intravesical chemotherapy lowers bladder recurrence rates and should be mandatory. Instillation of BCG and mitomycin C as monotherapy in the upper urinary tract can be considered in selected patients, keeping in mind the limited evidence and the risk of complications. Seen the preliminary but promising results, adjuvant platinum-based chemotherapy should be considered for patients with T2 + disease after RNU.

In general, there is very little evidence regarding all treatment modalities in UTUC. Further prospective randomized clinical trials are warranted in this area.
